# Supplementary material for: The Development of a Multiplex Real-Time Quantitative PCR Assay for the Differential Detection of the Wild-Type Strain and the MGF505-2R, EP402R and I177L Gene-Deleted Strain of the African Swine Fever Virus
Source: Animals (Basel). 2022 Jul 8;12(14):1754. doi: 10.3390/ani12141754 (PMC9311895; doi:10.3390/ani12141754)
Supplement: Supplementary file 1 [file animals-12-01754-s001.zip › animals-1774777-supplementary.pdf]

Table S1. The sequences of recombinant plasmids pASFV-ΔMGF505-2R, pASFV-ΔEP402R and pASFV-ΔI177L.

Plasmid	Sequence
p-ΔMGF505-2R	TATCCCTAAGAATATATCTTATAACTAGACTTATAGCAGTAAAAATCAACTTTGGTTATT<br>CTTTTTAATATAAAACGTCTAATTACTTGCAAAGGACTATAAAGCCCATTTTCCTCAGCT<br>AGAATTTTTATTTTTTAATGAAGTAGGGGGATAATTCAATAGATATCCATCATTAATATT<br>GATTATATTTTCGAATATTATCTTCTATGGTGCAAGATAATCATCTAGCGCGTGAAACAT<br>GTCCTCTTCTCTTCAGGAACTTTGTCGAAAAAAGCTGCCT
p-ΔEP402R	AATGAGTCAGTACTATTACGTGATAGTGGATATTACAAAAAACAAAAACATGTAAGTTT<br>ATTATATATTTGTAGTAAATAATATTTTTAATTACTTAAAATTTTTATATATAAGTTTTTG<br>ATACTATATTATAAAACATATGTTCATAAATATGTACTATATATTAATTATTTAACCTTTC<br>AAGCTGGTCTTCATTTAAATTTAAAATCCACTAATAAAATGTATTTTCTAGTAGCAGATC<br>ATCGAGAACATCATGTGATTCCTTTTCTTAAAACCGATTTCCATCACATGCATCAAAAT
p-ΔI177L	GGGATTCTCTATCAGGTGTCTGTACTCTGCTATTAAAAACCTGGAAACCATGGTTATTTA<br>ATATTAATTAAATTCCCTGGTTTATTCCTCCTTAAAAGTAGATGAACCTCTTTTGTTTTTT<br>ATTGGGTTCATTTTTACTAAATTTATGAATAAAAGATTATTATATTCGAATGTTTGTCCAA<br>TATGGACAACTTTGTCACCAGATGTTACATTTGATTTGGTTGTTAGTGGCTGAAGCTTGG<br>CACAATCAAAAATAAGCCCATTAACACTAAGATATAG
